# Measuring Viscosity and Consistency in Thickened Liquids for Dysphagia: Is There a Correlation Between Different Methods?

**DOI:** 10.3390/foods14132384

**Published:** 2025-07-05

**Authors:** Javier Marín-Sánchez, Sofía Gimeno-Ruiz, Alejandro Berzosa, Javier Raso, Cristina Sánchez-Gimeno

**Affiliations:** Food Technology, Facultad de Veterinaria, Instituto Agroalimentario de Aragón-IA2, Universidad de Zaragoza-CITA, 50013 Zaragoza, Spain; j.marin@unizar.es (J.M.-S.); gimenoruizsofia@gmail.com (S.G.-R.); aberzosa@unizar.es (A.B.); jraso@unizar.es (J.R.)

**Keywords:** dysphagia, thickened liquids, viscosity measurement, consistency, food rheology

## Abstract

Dysphagia is a common clinical condition, especially among older adults, associated with an increased risk of malnutrition, aspiration, and respiratory complications. A key therapeutic approach involves modifying liquid consistency using thickening agents to achieve safer swallowing. Although rotational rheometry offers accurate viscosity characterization, its complexity and cost limit routine application in clinical or domestic settings. This study evaluates and correlates different methods for measuring the viscosity of thickened liquids, comparing rheological data with empirical techniques such as the Ford cup, Bostwick consistometer, and Line-Spread Test (LST). Several thickeners were tested—guar gum, xanthan gum, a guar/xanthan blend, maltodextrin-based mixtures, and a commercial thickener—across a range of concentrations, temperatures, and preparation times. The results demonstrate that simple methods, particularly the Bostwick consistometer and LST, show strong correlations with rheometer measurements within the International Dysphagia Diet Standardisation Initiative (IDDSI) Level 2 (mildly thick) and Level 3 (moderately thick) ranges. However, limitations were observed at extreme viscosities, where certain methods lacked sensitivity or operational feasibility. These findings support the potential of empirical tools for practical viscosity screening in dysphagia management, especially where rheometry is unavailable. This work provides evidence-based guidance for clinicians, caregivers, and food service professionals seeking safe, reproducible, and standardized approaches to fluid consistency assessment.

## 1. Introduction

Dysphagia, or difficulty in swallowing, is a prevalent medical condition affecting individuals across various age groups, particularly the elderly and those with neurological disorders such as stroke, Parkinson’s disease, and dementia. The inability to swallow safely can lead to severe health complications, including malnutrition, dehydration, aspiration pneumonia, and increased mortality rates [1]. One of the primary therapeutic strategies for managing dysphagia is modifying the texture of foods and liquids to enhance swallowing safety and reduce aspiration risk [2]. In this context, thickened liquids are widely used as they slow down fluid transit through the oropharyngeal region, allowing for better airway protection [3,4,5]. However, achieving a consistent and standardized liquid thickness is challenging due to variations in thickener composition, preparation methods, and environmental factors such as temperature and time [6].

Thickening agents have evolved over time, transitioning from starch-based formulations to gum-based alternatives such as xanthan and guar gum. The advantages of gum-based thickeners include improved stability, solubility, and superior viscosity retention over time [2,7]. Starch-based thickeners, in contrast, are prone to enzymatic breakdown by salivary amylase, which can lead to a rapid decrease in viscosity and a subsequent increase in aspiration risk [5]. The efficacy of thickened liquids in dysphagia management is primarily determined by their rheological properties, particularly viscosity, which governs bolus flow behavior during swallowing [8]. An optimal viscosity ensures that the liquid flows at a controlled rate while remaining manageable for the patient. If too thin, it may increase aspiration risk; if too thick, it can lead to poor compliance and pharyngeal residue accumulation, exacerbating swallowing difficulties [1,9].

The role of viscosity in dysphagia management has been widely studied, demonstrating that increased viscosity provides greater bolus control, allowing individuals with impaired swallowing function more time to close the airway and initiate the swallowing reflex [10]. However, viscosity alone is not the sole determinant of safe swallowing; other properties such as bolus cohesiveness, elasticity, and surface tension also influence swallowing dynamics [11]. Therefore, a comprehensive assessment of thickened liquids must consider multiple rheological factors.

To characterize the rheological properties of thickened liquids, various methods have been employed. Rotational rheometry provides precise viscosity measurements under controlled shear conditions [8,12]. This technique is particularly useful for evaluating shear thinning behavior, a phenomenon where viscosity decreases with increasing shear rate, mimicking the conditions experienced during swallowing [13]. Despite its accuracy, rheometry requires specialized equipment and technical expertise, making it impractical for routine clinical or home care applications. Consequently, simpler methods such as the Bostwick consistometer and Ford cup, and Line Spread Test (LST) have been proposed as viable alternatives for assessing liquid consistency [3,14]. The Bostwick consistometer and LST measure the spread of a liquid over time, with greater spread indicating lower viscosity, whereas the Ford cup determines the time required for a liquid to flow through an orifice [11,15].

Previous studies have explored the relationship between viscosity measurement techniques, with some focusing exclusively on empirical methods [16,17] and others using rheological techniques [6,12,18,19,20,21]. Comparisons between viscometers/rheometers and rapid assessment tools such as the LST [22] and International Dysphagia Diet Standardisation Initiative (IDDSI) Flow Test [23,24,25] have also been reported. Furthermore, novel simple instruments for viscosity assessment have been evaluated [26,27]. While empirical methods provide practical and cost-effective solutions, their correlation with rheological measurements remains an area of ongoing investigation, particularly in extreme viscosity ranges where accuracy may be compromised [3].

One of the main limitations of empirical methods is their inability to fully capture the dynamic flow properties of thickened liquids during swallowing. For instance, the Bostwick consistometer measures the distance a liquid spreads in a fixed time, offering a useful but limited approximation of viscosity [28]. Similarly, the Ford cup is highly sensitive to liquid density and does not account for non-Newtonian behaviors [29]. Understanding how these methods correlate with rheological data is crucial for enhancing dysphagia management strategies.

Given the critical role of viscosity in dysphagia management, developing a reliable and accessible method for assessing thickened liquid consistency is essential. The objective of this study was to evaluate and correlate different viscosity measurement techniques, comparing rotational rheometry with alternative empirical methods. By analyzing various thickening agents under different concentrations, temperatures, and preparation conditions, this research aimed to determine the extent to which simple measurement tools can provide accurate and reproducible assessments of liquid consistency. Furthermore, this study sought to establish whether empirical methods can be reliably used in clinical and home settings where rheometry is not feasible.

## 2. Materials and Methods

### 2.1. Materials

Three types of powdered thickening agents were used: guar gum (GG), xanthan gum (XG), and maltodextrin (M) (HSN, Granada, Spain), as well as a commercial thickener (CT) containing maltodextrin, xanthan gum, and guar gum (Nutricia Nutilis Clear^®^, Liverpool, UK). The exact proportions of these ingredients are not disclosed by the manufacturer. A 1:1 blend of guar gum and xanthan gum (GG/XG) was also evaluated.

### 2.2. Sample Preparation

Thickened solutions were prepared by mixing distilled water and thickening agents at room temperature using a blender (Jocca, Zaragoza, Spain) for two minutes to ensure complete solubilization. The concentration ranges were established based on previous studies [30]. Gums were prepared at concentrations between 0.5% and 4% (*w*/*v*), while maltodextrin was used at concentrations ranging from 10% to 50%, either alone or in combination with gums. The commercial thickener was prepared following the manufacturer’s recommendations to achieve nectar-like, honey-like, and spoon-thick consistencies. These texture levels correspond to Levels 2, 3, and 4, respectively, as defined by the IDDSI framework [9].

### 2.3. Rheological Measurements

Rheological analyses were performed using a stress-controlled rheometer (MCR 301, Anton Paar Physica, Graz, Austria) equipped with a CC17 coaxial cylinder geometry. Unless otherwise specified, all measurements were conducted at 20 °C and a shear rate of 50 s^−1^, conditions selected to approximate those encountered during swallowing. This reference shear rate is consistent with previous studies on thickened liquids classified under the National Dysphagia Diet (NDD), where viscosity at 50 s^−1^ has been commonly used to define consistency levels [23].

To evaluate the effect of concentration on viscosity, samples were prepared using a range of concentrations from 0.5% to 4% for gum-based thickeners (GG, XG, and a 1:1 GG/XG blend). The commercial thickener (CT) was tested following the manufacturer’s instructions to obtain consistencies corresponding to IDDSI Levels 2 (nectar-like), 3 (honey-like), and 4 (spoon-thick). In the case of maltodextrin (M) and its mixtures with 0.5% GG or XG, higher concentrations (up to 40%) were required due to their lower thickening efficiency.

For the subsequent analyses—temporal stability, temperature dependence, and flow behavior—samples were prepared at concentrations that achieved a similar target viscosity of 0.5–0.6 Pa·s. This reference viscosity was reached using 1.0% GG, 2.0% XG, and 1.5% of the GG/XG blend. For the commercial thickener, the concentration corresponding to IDDSI Level 2 (1.5%) was selected as representative. Maltodextrin-based samples were excluded from these tests due to their inability to reach the reference viscosity range.

To assess temporal stability, viscosity measurements were recorded at 0, 2, 4, 6, and 24 h after preparation under the same reference conditions (20 °C, 50 s^−1^), simulating scenarios where thickened liquids are consumed with delay.

For the evaluation of temperature dependence, viscosity was measured at 5, 10, 15, 20, 25, 30, and 35 °C while maintaining a constant shear rate of 50 s^−1^. A digital thermometer with a data logger (Almemo 2590, Ahlborn, Holzkirchen, Germany) was used to monitor sample temperature during measurements.

Flow behavior was characterized by recording viscosity over a range of shear rates (8–100 s^−1^) at 20 °C. This analysis allowed for the identification of shear thinning behavior typical of non-Newtonian fluids used in dysphagia management [8].

### 2.4. Empirical Thickness Tests

#### 2.4.1. Line Spread Test

Measurements were performed using a cylindrical acrylic tube (height: 3.5 cm; diameter: 7.75 cm) placed on a Teflon board with concentric 1 cm markings (Ernesto, Beijing, China). The sample was poured into the tube, leveled with a spatula, and the tube was lifted simultaneously with the start of a timer. The spread diameter was recorded after 60 s at three perpendicular points. The results are expressed in centimeters (cm). All tests were conducted in triplicate.

#### 2.4.2. Bostwick Consistometer

A stainless-steel Bostwick consistometer (Endecotts, Cole-Parmer, London, UK), with a 0.5 cm graduated trough, was used. After leveling the device, 100 mL of sample was poured into the reservoir and smoothed with a spatula. Upon releasing the gate, the distance traveled by the sample after 30 s was measured in cm. Each measurement was performed in triplicate.

#### 2.4.3. Ford Cup

The Ford cup test was carried out using a stainless-steel Ford cup with a 6.0 mm orifice (Selecta, Barcelona, Spain), in accordance with the UNE-EN ISO 2431 standard [31]. This method is equivalent in principle to the IDDSI Flow Test, as it measures the time required for a given volume of liquid to flow through a standardized orifice under gravity. Before testing, the cup was leveled on a stable, draft-free surface using the device’s adjustable screws. The orifice was temporarily sealed, and the cup was filled with the sample, ensuring that it was bubble-free. A collection container was placed beneath the orifice, maintaining a clearance of at least 10 cm between the orifice and the sample surface in the container. Once the finger was removed, a stopwatch was simultaneously started. The flow time corresponded to the moment when the continuous stream of liquid broke. All measurements were performed in triplicate.

### 2.5. Statistical Analysis

Each determination was performed in triplicate, and the results are expressed as the mean ± standard deviation. Correlations between viscosity measurement methods were analyzed using GraphPad Prism^®^ 8.4.2 (GraphPad, San Diego, CA, USA). Pearson’s correlation coefficient was calculated with a 95% confidence interval, and linear regression analysis was conducted to assess the strength and direction of relationships between variables.

## 3. Results and Discussion

### 3.1. Effect of Concentration on Viscosity

**Figure 1:**
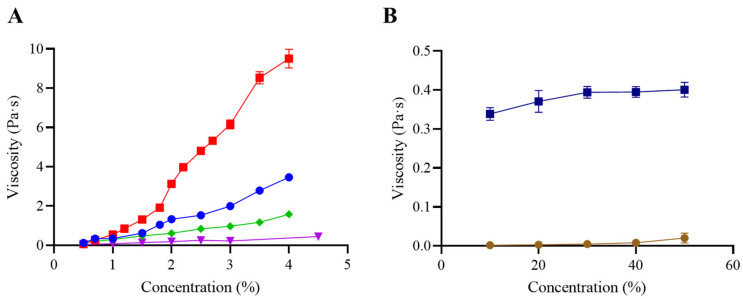
Viscosity measurement at different concentrations: (**A**) guar gum (■ GG), xanthan gum (⬥ XG), an equal mixture of guar and xanthan gum (● GG/XG), and the commercial thickener Nutricia Nutilis Clear (▼ CT); (**B**) maltodextrin (● M) and maltodextrin with 0.5% guar gum and 0.5% xanthan gum (■ M/GG/XG). Measurements were performed using a rheometer (MCR 301, Anton Paar Physica, Austria) at a shear rate of 50 s^−1^ and a temperature of 20 °C. Error bars represent the standard deviation from three independent sample preparations.

The effect of concentration on the viscosity of GG, XG, GG/XG, CT, and M was measured in a rotational rheometer (Figure 1A,B). Samples were tested across concentration ranges intended to encompass IDDSI-related textures (nectar, honey, and spoon-thick). For CT, the manufacturer’s recommended concentrations were also included. In the case of M, higher concentrations (10–50%) were required due to its lower thickening capacity in comparison with gums. To assess possible interactions, 0.5% of GG or XG was added to M formulations.

GG exhibited the strongest thickening effect, reaching a viscosity of 9.5 Pa·s at 4.0%. Viscosity also increased with concentration for XG and the GG/XG blend, although the maximum values were lower than those observed with GG alone. Notably, the blend showed slightly higher viscosity than XG by itself, suggesting a synergistic interaction between both gums. The increase in viscosity with concentration was previously described in other studies [6,20].

In contrast, M showed no significant increase in viscosity across the tested concentrations, and the values remained substantially lower than those of the gum-based samples. However, when GG or XG was incorporated into the formulation, viscosity increased markedly and followed a concentration-dependent trend, highlighting the limited thickening power of maltodextrin in isolation.

Viscosity of CT increased steadily with concentration. Although maltodextrin is listed as the main ingredient, the thickening performance is likely driven by the presence of gums such as GG and XG. These findings emphasize the relevance of formulation composition beyond the primary listed component when evaluating thickening behavior.

These findings are consistent with previous studies. Gum-based thickeners have been shown to exhibit a predictable and linear increase in viscosity with concentration, in contrast to starch-based or maltodextrin-containing products, which show exponential or inconsistent behavior [32]. This aligns with the results obtained for M and CT in the present study, where gum-based samples provided more stable and effective viscosity control across the tested concentrations.

Moreover, the poor performance of M when used alone supports earlier evidence indicating that maltodextrin lacks sufficient thickening power to meet clinical viscosity targets without the addition of hydrocolloids. The improved behavior of M upon addition of GG or XG also reinforces the role of gums in achieving adequate rheological properties for safe swallowing.

Table 1 presents the classification of the tested samples according to the viscosity ranges defined by the National Dysphagia Diet Task Force (NDD) [33]. Each thickener exhibited distinct behavior across concentrations. GG showed high thickening efficiency, achieving pudding-like viscosity at 2.4%, while XG and GG/XG required higher concentrations to reach equivalent levels. In contrast, M remained within the thin range, even at concentrations up to 50%, highlighting its limited capacity to achieve therapeutic viscosity levels when used alone. However, the incorporation of GG or XG into M significantly improved its thickening performance, reaching honey-like consistencies at 40–50%.

For the commercial thickener (CT), the manufacturer’s recommended concentration of 3%—typically associated with a honey-like texture—yielded viscosity values corresponding instead to nectar-like levels under the conditions tested. Spoon-thick viscosity was only approached at 4.5%, suggesting a deviation from the expected classification. This underscores the importance of validating manufacturer guidelines under controlled rheological conditions to ensure therapeutic efficacy and patient safety.

Even small variations in viscosity within the same IDDSI level can be perceived by elderly individuals, particularly in the nectar and honey ranges [34]. This further emphasizes the clinical relevance of accurately adjusting and validating thickener concentrations to ensure consistency with target IDDSI levels. The underperformance of CT at the recommended 3% concentration—falling below expected viscosity—suggests a potential risk of under-thickening in real-life applications, and supports the need for precise rheological assessment in clinical protocols.

### 3.2. Effect of Time After Sample Preparation on Viscosity

In clinical practice, texture-modified liquids are often not consumed immediately after preparation. In many cases, they are prepared in advance—sometimes several hours before ingestion—especially in institutional or home settings [35]. This delay between preparation and consumption can lead to changes in viscosity, particularly in formulations based on traditional starch thickeners, which are prone to enzymatic degradation by salivary amylase and retrogradation phenomena. Such alterations may result in a final viscosity lower than the intended therapeutic level, potentially increasing the risk of aspiration and reducing treatment efficacy. In contrast, new-generation gum-based thickeners, such as those evaluated in this study, tend to exhibit greater stability over time, offering a more reliable solution for dysphagia management. Therefore, evaluating the time-dependent behavior of these formulations is essential to ensure clinical safety and consistency [5].

**Figure 2:**
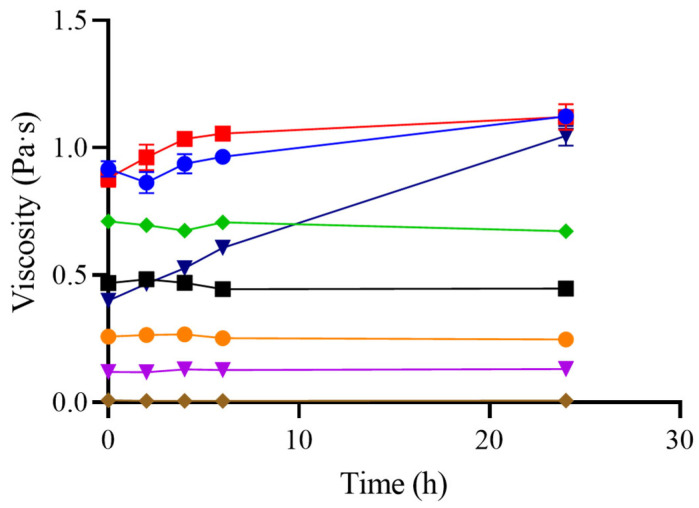
Viscosity measurement of different thickeners over time after preparation. The following formulations were used: 1% guar gum (■ GG), 2% xanthan gum (⬥ XG), an equal mixture of guar and xanthan gum (● GG/XG), a commercial thickener at the manufacturer’s recommended concentrations (▼ CT 1.5, ● 3, and ■ 4.5 %), 40% maltodextrin (⬥ M), and maltodextrin with 0.5% guar gum and 0.5% xanthan gum (▼ M/GG/XG). Measurements were performed using a rheometer (MCR 301, Anton Paar Physica, Austria) at a shear rate of 50 s^−1^ and a temperature of 20°C. Error bars represent the standard deviation from three independent sample preparations.

Figure 2 shows the evolution of viscosity at 25 °C over a 24 h period for the different formulations. An increase in viscosity over time was observed for GG, GG/XG, and M/GG/XG formulations. In the case of M/GG/XG, viscosity nearly doubled after 24 h, indicating a significant structural evolution post-mixing. This phenomenon has been reported previously for guar gum-based thickeners, where hydration continues gradually and can result in a progressive viscosity increase [20].

In contrast, no significant change in viscosity was observed for XG or M alone, suggesting that the increase is likely attributable to the presence of GG. XG tends to hydrate rapidly and reaches its viscosity peak shortly after preparation, while GG may require a longer hydration period.

The commercial thickener (CT) showed stable viscosity over 24 h, despite manufacturer recommendations suggesting consumption within 2 h. CT contains maltodextrin, xanthan gum, and guar gum (in decreasing order), and the low proportion of GG may explain the absence of time-dependent viscosity increases. Similar findings were reported in a previous study, which observed only slight viscosity increases within the first 3 h after mixing [12]. In our study, even over extended periods, CT viscosity remained relatively unchanged, resembling the behavior of XG alone, likely due to the higher proportion of this gum in the formulation.

Maltodextrin is incorporated in CT formulations not only to improve dispersibility in water, but also to serve as a caloric supplement, supporting nutritional needs in patients at a risk of malnutrition. Thickeners combining maltodextrin and gums are sometimes referred to as “new-generation” thickeners, designed to provide viscosity stability under varying conditions, including extended holding times [7].

This behavior contrasts with traditional starch-based thickeners, which typically show an increase in viscosity over time due to continued starch hydration and swelling. However, such systems are also susceptible to enzymatic breakdown in the oral cavity by salivary α-amylase, leading to a rapid drop in viscosity during swallowing and a potentially increased risk of aspiration [6]. From this perspective, gum-based and gum-enriched formulations offer a more reliable option in terms of both rheological stability and clinical safety.

### 3.3. Effect of Temperature on Viscosity

**Figure 3:**
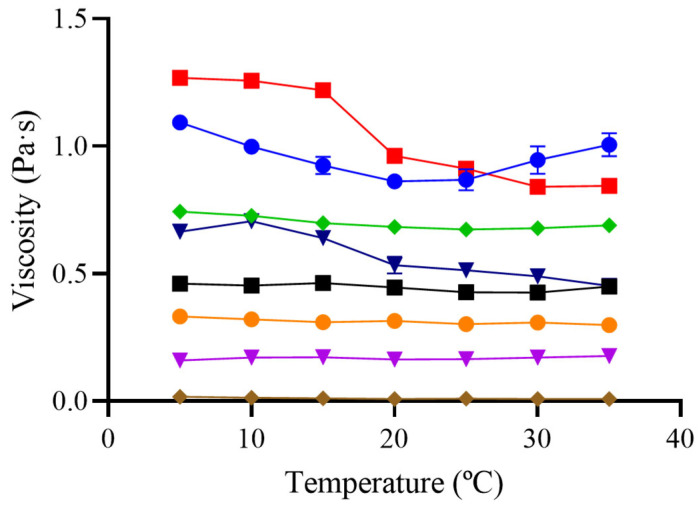
Viscosity measurement of the thickeners used in this study at different temperatures. The following formulations were tested: 1% guar gum (■ GG), 2% xanthan gum (⬥ XG), an equal mixture of guar and xanthan gum (● GG/XG), a commercial thickener at the manufacturer’s recommended concentrations (▼ CT 1.5, ● 3, and ■ 4.5%), 40% maltodextrin (⬥ M), and maltodextrin with 0.5% guar gum and 0.5% xanthan gum (▼ M/GG/XG). Measurements were performed using a rheometer (MCR 301, Anton Paar Physica, Austria) at a shear rate of 50 s^−1^. Error bars represent the standard deviation from three independent sample preparations.

As shown in Figure 3, GG and M/GG/XG exhibited an inverse relationship between temperature and viscosity, consistent with this expected behavior. In both cases, viscosity decreased progressively between 25 °C and 45 °C, indicating thermal sensitivity likely driven by the presence of guar gum. The GG/XG blend displayed a slight viscosity increase between 25 °C and 35 °C, followed by stabilization.

This anomalous increase in viscosity upon mild heating has been previously reported for systems containing xanthan gum, and is often attributed to the enhanced alignment and interaction of its polymer chains. Xanthan gum possesses a rigid, rod-like molecular structure that facilitates intermolecular associations under certain thermal conditions, particularly in combination with other gums such as guar. The presence of guar gum may further promote network formation due to synergistic effects, resulting in a non-linear increase in viscosity. This phenomenon, sometimes referred to as thermally induced gelation or entanglement enhancement, has been described in gum-based systems where mild heating enhances viscosity before any thermal degradation occurs [15].

In contrast, XG, M, and CT showed negligible changes in viscosity across the temperature range studied. The thermal stability of XG is well documented and attributed to its rigid molecular conformation, which maintains its rheological properties even under moderate heating. Similarly, the absence of thermal response in CT is likely explained by the dominance of XG in its composition, despite the presence of maltodextrin and GG in smaller proportions. The behavior of M was also stable, but its very low baseline viscosity limits its practical relevance in isolation.

### 3.4. Flow Behavior

**Figure 4:**
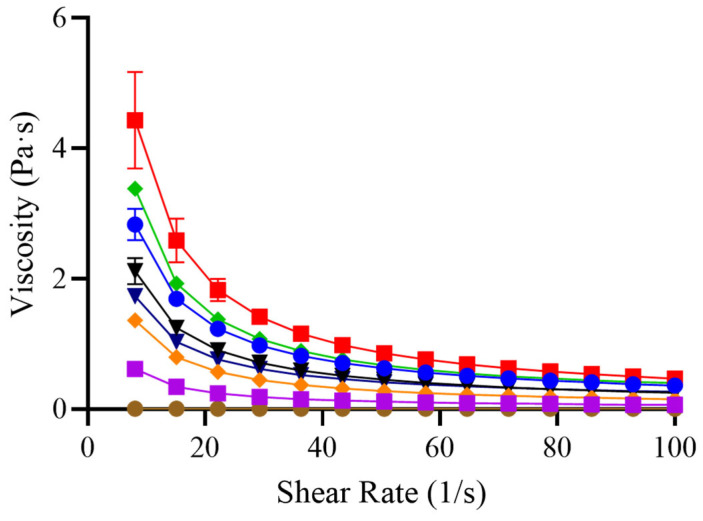
Viscosity of different thickeners as a function of shear rate. The following formulations were tested: 1% guar gum (■ GG), 2% xanthan gum (⬥ XG), an equal mixture of guar and xanthan gum (● GG/XG), a commercial thickener at the manufacturer’s recommended concentrations (■ CT 1.5, ⬥ 3, and ▼ 4.5 %), 40% maltodextrin (● M), and maltodextrin with 0.5% guar gum and 0.5% xanthan gum (▼ M/GG/XG). Measurements were performed using a rheometer (MCR 301, Anton Paar Physica, Austria) at a temperature of 20 °C. Error bars represent the standard deviation from three independent sample preparations.

The flow behavior of the thickening agents is shown in Figure 4. Apparent viscosity was measured as a function of shear rate to characterize the rheological profile of each sample. Particular attention was given to the viscosity at 50 s^−1^, a shear rate commonly used as a physiological reference for swallowing, as it approximates the conditions in the oropharynx during bolus transit [15].

GG, XG, and CT exhibited pseudoplastic (shear thinning) behavior, with viscosity decreasing as shear rate increased. This behavior is advantageous for individuals with dysphagia: at rest, higher viscosity provides bolus control and reduces the risk of premature spillage into the airway, while under the shear forces generated during swallowing, the viscosity drops, facilitating a smoother transit through the pharynx. Similar pseudoplastic profiles have been reported for a range of gum-based and commercial thickeners [6,12,23], and are considered a key property for ensuring both safety and efficacy in texture-modified liquids. This dual behavior—characterized by high viscosity at rest and reduced resistance under shear—is fundamental for clinical use in dysphagia management. It ensures that the fluid remains cohesive and stable in the oral cavity, minimizing the risk of premature flow, while simultaneously allowing for a smooth and safe bolus transit during swallowing.

In contrast, M demonstrated Newtonian behavior, maintaining a constant viscosity regardless of shear rate. This rheological profile is intrinsic to the molecular structure of maltodextrin, a low-molecular-weight carbohydrate composed of short, linear chains that do not entangle or form structured networks in solution. As a result, maltodextrin lacks the viscoelastic properties required to develop a cohesive bolus. Previous studies have shown that maltodextrin-based systems exhibit low viscosity and poor viscoelastic structure, making them unsuitable as stand-alone thickening agents for dysphagia management [7,36]. Therefore, in clinical practice, maltodextrin should not be used alone in the formulation of thickened fluids for patients with swallowing disorders; it must be combined with structurally functional thickeners such as guar or xanthan gum to ensure safe and effective viscosity levels.

From a clinical perspective, the shear thinning behavior observed in gum-based and commercial thickeners is desirable, as it ensures that the fluid maintains sufficient thickness at rest while becoming easier to swallow under physiological shear. This adaptative flow behavior supports safe and efficient bolus transit, especially in patients with impaired swallowing reflexes [6]. In contrast, the Newtonian profile of maltodextrin reinforces its limited utility in dysphagia diets when used alone. These differences in flow behavior underline the importance of selecting thickeners not only based on concentration but also on their rheological response under dynamic conditions relevant to swallowing [8,12].

Among the gum-based thickeners evaluated, guar gum (GG) exhibited the highest viscosity at equivalent concentrations, which has important practical implications for institutional settings. Specifically, achieving a target viscosity with GG requires lower concentrations compared to other thickeners, allowing for reduced dosage and potentially improving texture perception and patient compliance. Additionally, formulations based on GG, or blends with xanthan gum (XG), demonstrated stable rheological properties over time and under varying temperatures. Although GG alone showed a slight decrease in viscosity with increasing temperature, this limitation was mitigated when combined with XG, as in the commercial thickener tested. This synergistic effect supports the formulation of blends that optimize thickening performance while maintaining functional stability.

### 3.5. Correlation Between Viscosity Measurement Methods

After the rheological characterization of the samples, alternative, simpler methods for estimating liquid consistency were evaluated and compared to the values obtained using a rotational rheometer.

In the case of the maltodextrin-based sample, the viscosity was too low to be reliably measured with the alternative methods. Therefore, only rheological measurements were possible, and no correlation analyses could be established for this material. Pearson’s correlation coefficient (r) was used to assess the linear relationship between viscosity measurements obtained by different methods.

**Figure 5:**
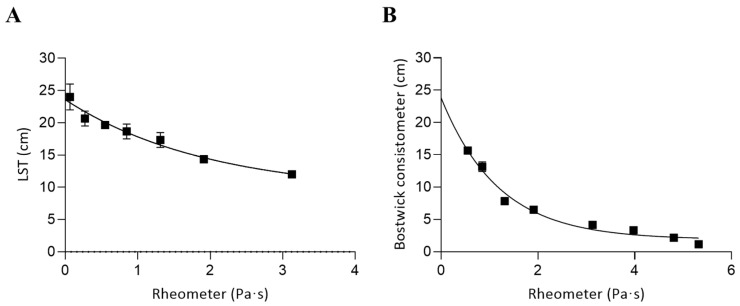
Correlation between the viscosity measured using a rheometer and alternative methods in guar gum solutions: (**A**) Line Spread Test and (**B**) Bostwick consistometer. Concentration ranges were 0.5–2.0% (**A**) and 1.0–2.7% (**B**). Error bars represent the standard deviation from three independent sample preparations.

Figure 5 shows the correlation between the viscosity of guar gum (GG) solutions measured with a rheometer and two empirical tests: the Line Spread Test (A) and the Bostwick consistometer (B). In this case, Ford cup measurements were not feasible due to the high viscosity of GG solutions, which exceeded the operational range of that method.

For the Line Spread Test, the best fit was obtained using the following nonlinear (one-phase decay) model:

$$Y=14.495\cdot e^{-0.5064X}+9.095$$

For the Bostwick consistometer, the corresponding equation was

$$Y=21.914\cdot e^{-0.8452X}+1.876$$

where *Y* represents the distance traveled by the sample in centimeters—either after 60 s in the Line Spread Test or after 30 s in the Bostwick consistometer—while *X* corresponds to the apparent viscosity (Pa·s) measured using a rotational rheometer.

For the Line Spread Test, applied to concentrations between 1.0% and 2.7%, a significant negative correlation was observed with viscosity values obtained from the rheometer, reflecting the expected inverse relationship between flow distance and viscosity. The data were best described by an exponential decay model with a strong fit (adjusted R^2^ = 0.916). When using the Bostwick consistometer at lower concentrations (0.5–2.0%), the correlation followed a similar nonlinear pattern, also showing a strong fit (adjusted R^2^ = 0.916). These results confirm that both empirical methods provide reliable estimates of relative consistency in GG solutions within their applicable concentration ranges.

A strong correlation was also found between the two empirical methods themselves (r = 0.93), supporting findings from previous studies that report similar predictive performance of distance-based methods for consistency estimation [3]. These approaches, although less precise than rheometry, offer valuable information when rapid or low-cost assessments are needed, particularly in clinical environments.

**Figure 6:**
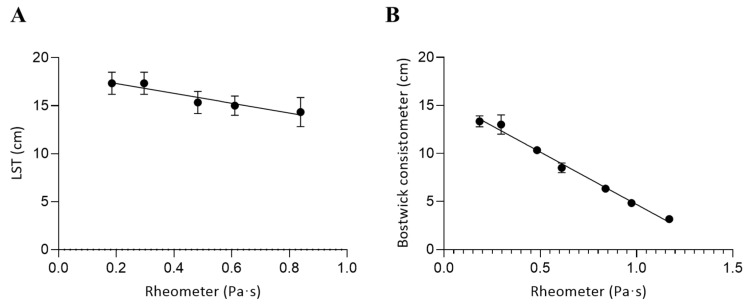
Correlation between the viscosity measured using a rheometer and alternative methods in xanthan gum solutions: (**A**) Line Spread Test and (**B**) Bostwick consistometer. Concentration ranges were 0.8–2.5% (**A**) and 0.8–4.0% (**B**). Error bars represent the standard deviation from three independent sample preparations.

The correlation between viscosity measurements obtained with a rheometer and those derived from empirical methods in xanthan gum solutions is presented in Figure 6. As in the case of GG, the Ford cup was not used because the high viscosity of XG solutions exceeds the measurement capacity of the instrument.

In this case, the best fit for both empirical methods was obtained using simple linear regression models, reflecting a direct and proportional relationship between viscosity and flow distance.

Line Spread Test:

Y=−5.127·X+18.34

Bostwick consistometer:

Y=−10.90·X+15.59

where *Y* represents the distance traveled by the sample in centimeters—either after 60 s in the Line-Spread Test or after 30 s in the Bostwick consistometer—while *X* corresponds to the apparent viscosity (Pa·s) measured using a rotational rheometer.

In the concentration range of 0.8–4.0%, the Bostwick consistometer showed an almost perfect negative correlation with the viscosity measured by rheometry (r = −0.99, *p* < 0.05). This indicates that the Bostwick test is highly reliable for estimating relative viscosity changes in XG solutions within this concentration range. When the Line Spread Test was applied to lower concentrations (0.8–2.5%), corresponding approximately to nectar- and honey-thick textures, a similarly strong correlation was observed (r = −0.95, *p* < 0.05), also statistically significant.

Additionally, the correlation between the two empirical methods themselves was high (r = 0.96), in agreement with previous studies showing that distance-based tests tend to provide consistent estimations of flow behavior in gum-thickened fluids [3,15]. These findings support the utility of the Bostwick consistometer and Line Spread Test as practical tools for rapid consistency assessment, particularly when used within their optimal viscosity ranges.

**Figure 7:**
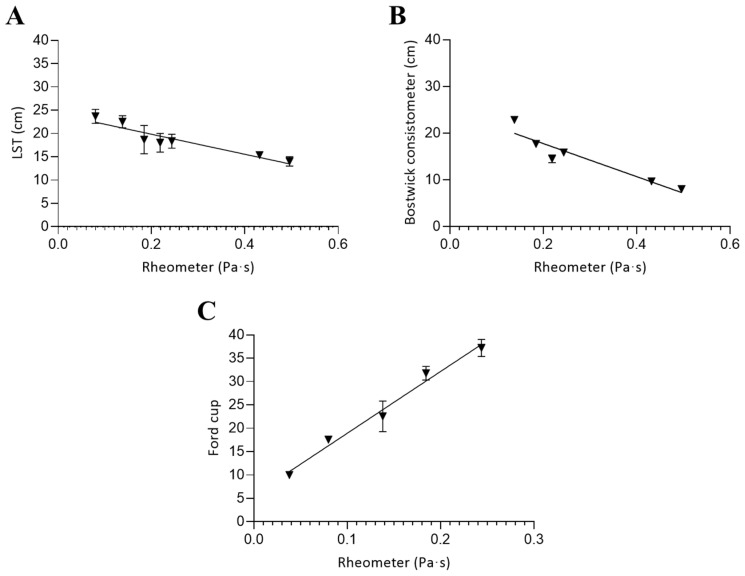
Correlation between the viscosity measured using a rheometer and alternative methods in commercial thickener solutions: (**A**) Line Spread Test, (**B**) Bostwick consistometer, and (**C**) Ford cup. Concentration ranges were 1.0–6.0% (**A**), 1.5–8.5% (**B**), and 0.5–2.5% (**C**). Error bars represent the standard deviation from three independent sample preparations.

The correlation between viscosity values measured with a rheometer and those obtained using alternative methods for the commercial thickener (CT) is shown in Figure 7. In this case, all three empirical techniques were applicable, including the Ford cup. Due to the relatively low viscosity of CT, the Ford cup could be used successfully—unlike in the gum-based formulations—particularly at concentrations corresponding to thin and nectar-like textures.

For the CT, viscosity values obtained using empirical methods showed strong linear correlations with those measured by rheometry. For the Line Spread Test, the corresponding regression equation was

Y=−21.49·X+24.14

where *Y* is the distance traveled (cm) after 60 s and *X* the apparent viscosity (Pa·s).

For the Bostwick consistometer, the corresponding equation was

Y=−35.43·X+24.86

with *Y* representing the distance after 30 s.

In the case of the Ford cup, which measures flow time until stream breakage, the corresponding equation was

Y=132.4·X+5.715

where *Y* is the time in seconds.

For the Bostwick consistometer, applied in the 1.5–8.5% concentration range, a strong negative correlation was observed with rheometer data (r = −0.92, *p* < 0.05). Although the Line Spread Test and flow-based methods have been previously evaluated in dysphagia research, the use of the Bostwick consistometer appears to be scarcely documented in this specific context. Its inclusion in this study offers new insights into its potential applicability for assessing consistency in commercial thickeners.

The Line Spread Test, used between 1.0% and 6.0%, showed a slightly higher correlation (r = −0.94, *p* < 0.05), supporting its effectiveness for the routine evaluation of moderately thick liquids. The highest correlation was observed between rheometer measurements and the Ford cup (r = −0.99, *p* < 0.05), within the 0.5–2.5% concentration range. This range corresponds to thin and nectar-like textures, approximately below 0.35 Pa·s according to the National Dysphagia Diet Task Force (NDD) classification.

The Ford cup also showed strong agreement with the other empirical methods, consistent with recent reports describing the utility of similar viscosity measuring devices for dysphagia diets [22,26]. These results reinforce the applicability of such tools for screening consistency, particularly in low-viscosity ranges where flow time methods are more sensitive.

Previous studies have also confirmed strong correlations between rheological data and alternative tests such as the Line Spread method and IDDSI flow test in commercial thickeners [3,6,15]. In the present work, CT demonstrated predictable and stable rheological behavior across methods, likely due to its formulation based on xanthan and guar gums, which enables consistent flow properties even when evaluated using simplified techniques.

Each of the evaluated empirical methods presents specific advantages and limitations depending on the viscosity range and type of thickening agent. The Ford cup proved to be the most limited in scope. Due to its design and operating principle based on flow time under gravity, it was only applicable in low-viscosity samples, specifically, in the commercial thickener (CT) at concentrations corresponding to thin and nectar-like textures (0.051–0.35 Pa·s). In gum-based formulations (GG, XG), the high viscosity prevented flow through the orifice, while in maltodextrin-based liquids, viscosity was too low to obtain meaningful measurements. Although the Ford cup showed excellent correlation with rheological data in the valid range, its applicability is restricted to a narrow viscosity window, limiting its utility for broader texture classification.

The Bostwick consistometer was more versatile and effective, especially for samples in the nectar and honey ranges. It provided consistent and statistically significant correlations with rheometer data for GG, XG, and CT, confirming its reliability in clinical or production environments where rapid assessments are needed. However, the method was not suitable for very low-viscosity samples such as maltodextrin solutions, nor for high-viscosity (spoon-thick) formulations, as the initial impulse required to start flow was insufficient.

The Line Spread Test also demonstrated utility for samples within intermediate viscosity ranges but showed greater variability in measurements. The irregular advance of the liquid across the plate led to heterogeneous spread patterns, requiring the use of multiple measurement points. Despite this, it allowed for differentiation between textures for GG, XG, and CT within appropriate concentration ranges. Its application was limited at the lower end (maltodextrin and diluted gums) due to insufficient structure and at the higher end (spoon-thick) due to minimal flow. The design of the spread test plate, with a measurement range between 12 cm and 26 cm, effectively confines its use to nectar- and honey-like textures. Similar methodological constraints have been reported in previous studies comparing the Line Spread Test performance with instrumental viscosity values [14].

Although these empirical methods are inherently less precise than rheometric techniques, their value lies in their accessibility, simplicity, and adaptability to real-world clinical settings. In the context of dysphagia management, where the rapid and reproducible assessment of liquid consistency is essential, tools such as the Ford Cup, Bostwick consistometer, and Line Spread Test offer practical alternatives for screening and routine monitoring. Their ability to estimate viscosity within defined concentration ranges allows for the consistent implementation of texture-modified diets, particularly in environments where specialized instrumentation is unavailable. These findings reinforce the idea that empirical methods, when properly calibrated and validated, can serve as reliable tools to support safe swallowing and improve the quality of care in institutional and home-based contexts.

## 4. Conclusions

This study demonstrated that simplified empirical methods—Ford cup, Bostwick consistometer, and Line Spread Test—can provide valuable and accessible tools for estimating the consistency of thickened liquids used in dysphagia management. When applied within their optimal operating ranges, these methods showed strong correlations with apparent viscosity values obtained from rotational rheometry at 50 s^−1^. This highlights their potential to serve as screening techniques in clinical, institutional, or home settings where access to rheological instrumentation is limited.

Each empirical method displayed specific advantages depending on the viscosity range. The Ford cup was especially effective for low-viscosity (nectar-like) fluids, while the Bostwick consistometer and Line Spread Test offered better performance in moderately thick and honey-like formulations. Although these methods lack the precision and dynamic profiling of a rheometer, their simplicity, affordability, and reproducibility make them suitable for routine quality control of texture-modified liquids.

These findings support the integration of empirical tests into dysphagia care protocols, provided that their limitations are understood and validated concentration-to-viscosity benchmarks are established. Future research should explore a wider range of commercial products and food matrices, including fruit juices and milk-based beverages. In addition, evaluating viscosity at physiological (oral) temperatures would provide further insight into the in-mouth behavior of thickened liquids, enhancing the clinical applicability of simplified measurement techniques. Furthermore, future work should consider extending rheological measurements to a broader shear rate range, beyond the standard 50 s^−1^, in order to better reflect the dynamic conditions experienced during the various phases of swallowing.

## Figures and Tables

**Table 1:** Classification of the concentrations (%) of the thickeners used in this study according to viscosity levels defined by the National Dysphagia Diet Task Force (NDD) [33].

IDDSI Level	Level 0	Level 1–2	Level 3	Level 4
Description	Thin	Slightly thick/Nectar-like	Moderately thick/Honey-like	Extremely thick/Pudding-like
Viscosity range (Pa·s)	0.001–0.050	0.051–0.350	0.351–1.750	>1.751
GG		0.5	1.0–1.5	2.4
XG		0.5–1.0	1.5–4.0	
GG/XG		0.5–1.0	1.5–2.0	2.5–4.0
M	10.0–50.0			
M/GG/XG		10.0	40.0–50.0	
CT	0.5	1.0–4.0	4.5–8.5	

## Data Availability

Data will be made available on request.
